# The effects of nonspecific HIF1*α* inhibitors on development of castrate resistance and metastases in prostate cancer

**DOI:** 10.1002/cam4.189

**Published:** 2014-01-27

**Authors:** Weranja K B Ranasinghe, Shomik Sengupta, Scott Williams, Mike Chang, Arthur Shulkes, Damien M Bolton, Graham Baldwin, Oneel Patel

**Affiliations:** 1 Department of Urology, Austin Health/University of MelbourneHeidelberg, Victoria, Australia; 2 Department of Surgery, Austin Health/University of MelbourneHeidelberg, Victoria, Australia; 3 Royal Melbourne HospitalMelbourne, Australia; 4 Peter MacCallum Cancer InstituteMelbourne, Australia

**Keywords:** Castrate resistance, Hypoxia-inducible factor, inhibitors, metastases, prostate cancer

## Abstract

Expression of hypoxia-inducible factor (HIF)1*α* increases the risk of castrate-resistant prostate cancer (CRPC) and metastases in patients on androgen deprivation therapy (ADT) for prostate cancer (PC). We aimed to investigate the effects of nonspecific HIF1*α* inhibitors (Digoxin, metformin, and angiotensin-2 receptor blockers) on development of CRPC and metastases while on ADT. A retrospective review of prospectively collected medical records was conducted of all men who had continuous ADT as first-line therapy for CRPC at the Austin Hospital from 1983 to 2011. Association between HIF1*α* inhibitor medications and time to develop CRPC was investigated using actuarial statistics. Ninety-eight patients meeting the criteria were identified. Eighteen patients (21.4%) were treated with the nonspecific HIF1*α* inhibitors. Both groups had similar characteristics, apart from patients on HIF1*α* inhibitors being older (70 years vs. 63.9 years). The median CRPC-free survival was longer in men using HIF1*α* inhibitors compared to those not on inhibitors (6.7 years vs. 2.7 years, *P* = 0.01) and there was a 71% reduction in the risk of developing CRPC (HR 0.29 [95% CI 0.10–0.78] *P* = 0.02) after adjustment for Gleason score, age, and prostate-specific antigen (PSA). The median metastasis-free survival in men on HIF1*α* inhibitors was also significantly longer compared to those on no inhibitors (5.1 years vs. 2.6 years, *P* = 0.01) with an 81% reduction in the risk of developing metastases (HR 0.19 [CI 0.05–0.76] *P* = 0.02) after adjustment for Gleason score, age, and PSA. Nonspecific HIF1*α* inhibitors appear to increase the progression-free survival and reduce the risk of developing CRPC and metastases in patients on continuous ADT.

## Introduction

Androgen deprivation therapy (ADT) is a standard treatment for advanced prostate cancer (PC). However, the disease progresses and castrate-resistant prostate cancer (CRPC) develops in a significant proportion of men while on ADT 1. CRPC is a lethal form of PC that progresses and metastasizes with a median survival of 1–3 years 2. In some series greater than 84% of CRPC patients have metastases at diagnosis, while a further 33% of patients who do not have metastases at the diagnosis of CRPC may be expected to develop metastases within 2 years 3. Treatment of CRPC can be difficult as the majority of these tumors become unresponsive to chemotherapy and radiotherapy. Thus, novel therapeutic strategies targeted at delaying or preventing CRPC are of crucial importance in combating this disease.

Currently, there is no accepted method to determine which PCs will develop resistance to androgen deprivation. Furthermore, to our knowledge no current agents have been shown to prevent or delay development of castrate resistance. We previously demonstrated that the expression of hypoxia-inducible factor 1*α* (HIF1*α*) increases the risk of CRPC and metastases in patients on ADT for PC 4, and therefore hypothesized that inhibition of HIF1*α* may delay or prevent development of these adverse consequences.

To overcome the exorbitant costs of new drug development and to speed up the discovery of novel drug targets, one approach is to find drugs with well-established toxicologic, pharmacokinetic, and pharmacodynamic profiles that may be effective against an unrelated indication. Digoxin, metformin, and angiotensin-2 receptor blockers (ARB) are three commonly used cardiovascular medications, which have all been shown to inhibit HIF1*α* by different mechanisms 5–7. The effects of these nonspecific HIF1*α* inhibitors on the development of CRPC and metastases were therefore investigated in this study.

## Patients and Methods

### Patient characteristics

A retrospective analysis was performed on a prospectively maintained database of all men who received chemotherapy subsequent to CRPC development and who were previously treated with continuous ADT at the Austin Hospital, Melbourne, Australia from 1983 to 2011. ADT as primary or salvage therapy following radiotherapy was allowed. Drug histories were obtained from the hospital records to identify men being treated with the nonspecific HIF1*α* inhibitors: digoxin, metformin, and ARBs at the time of starting ADT. All outcome and pathological data were obtained through the Victoria Biobank and the Department of Anatomical Pathology at the Austin Hospital, Victoria, Australia. Approval for this study was obtained from the Austin Health Human Research Ethics Committee.

CRPC was defined as two consecutive rises of prostate-specific antigen (PSA) of over 50% PSA nadir at least 1 week apart while on ADT. Time to CRPC was determined from the date of initiation of primary ADT or any form of salvage ADT to the date of the second PSA rise. Patients without metastatic disease at the time of commencing ADT were eligible for analysis of time to metastases. In this case, patients were investigated at their physician's discretion, and the date of initial identification of metastatic disease defined the endpoint.

### Immunohistochemistry

All available paraffin-embedded tissue samples were obtained for this cohort of men and were stained for HIF1*α* using a previously published protocol 4.

### Statistics

The Wilcoxon and Mann–Whitney *U* tests were used to compare any differences in characteristics between the patient groups (those on HIF1*α* inhibitors vs. those not). CRPC-free survival and metastasis-free survival were assessed using the Kaplan–Meier method and log-rank test. The impact of HIF1*α* inhibition, age, Gleason score, and PSA at the time of starting ADT on outcomes was assessed using Cox proportional hazards regression. Two-sided *P*-values were utilized, and a *P*-value of <0.05 was considered statistically significant. All analyses were performed with SPSS software (version 17.0; SPSS Inc, Chicago, IL).

## Results

Ninety-eight patients meeting the criteria were identified of whom 81 received ADT as primary therapy and 17 received ADT for biochemical recurrence post radiotherapy. Thirty-eight men (45%) were excluded from the time to metastases analysis as they had developed bony metastases prior to starting ADT.

Eighteen patients (21.4%) were on nonspecific HIF1*α* inhibitors prior to androgen deprivation. Of this cohort, there were eight patients on metformin, nine on digoxin, and four on ARBs (irbesartan (3/4) and candesartan (1/4). Three patients were on dual inhibitors with one person in each pair of digoxin and metformin, digoxin and ARB, and metformin and ARB. The dosages used were standard with six out of eight men on metformin on 500 mg twice daily and two on 1 g twice daily. All men on digoxin had 62.5 *μ*g daily, all men on irbesartan had 300 mg daily, and one man on candesartan 16 mg daily. On analysis of the baseline patient characteristics (Table 1), both groups had similar median Gleason scores (7.5 and 8). The median time of follow-up in both groups was also similar. However, the patients on HIF1*α* inhibitors were significantly older (70 years vs. 63.9 years, *P* = 0.009). Although both the median PSA score and the proportion of D'Amico high-risk disease (46% vs. 59%) were lower in the patients on HIF1*α* inhibitors, there was no statistically significant difference, apart from age, between any of these variables in the two groups.

**Table 1:** Patient characteristics.

	HIF1*α* inhibitors	No HIF1*α* inhibitors	*P*-value
No of patients	18	66	
Average age	70 (54–84)	63.9 (44–81)	0.009
Median Gleason score	7.5 (6–9)	8 (6–10)	0.747
Median PSA at start of androgen deprivation	97 (0–5705)	407 (0–4780)	0.08
Percentage D'Amico high-risk disease (%)	46	59	0.50
Median follow-up (years)	3.2 (0.34–9.5)	2.3 (1.0–8.5)	0.099

HIF, hypoxia-inducible factor; PSA, prostate-specific antigen. The baseline characteristics of the patients with and without HIF1*α* inhibitor treatment are shown. Differences between the two groups were analyzed using Wilcoxon and Mann–Whitney *U* tests and *P*-values were calculated. The range is demonstrated within brackets.

### HIF1*α* expression in tumors of men on nonspecific HIF1*α* inhibitors

**Figure 1:**
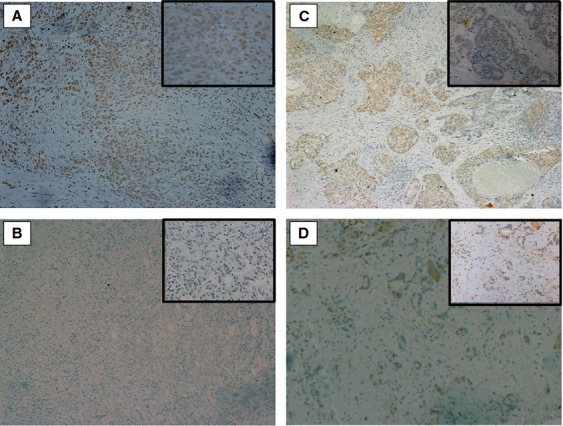
Hypoxia-inducible factor (HIF)1*α* expression is lower in tumors from men on metformin. All available tissue samples were stained immunohistochemically for HIF1*α* protein, and compared with samples matched for Gleason score. HIF1*α* expression was greater in trans urethral resection of prostate (TURP) specimens (A, C) from men who had Gleason 5 + 4 tumors when compared with samples from men on metformin with tumors of the same Gleason score (B, D). Inset magnification: 20×.

Tissue samples were available for 28 men, but of these only four had been on nonspecific HIF1*α* inhibitors (metformin in all cases). There was an obvious reduction in HIF1*α* expression in the samples from men on metformin (Fig. 1), when compared with matched samples from men with similar Gleason score, age, and procedure.

### Time to CRPC

**Figure 2:**
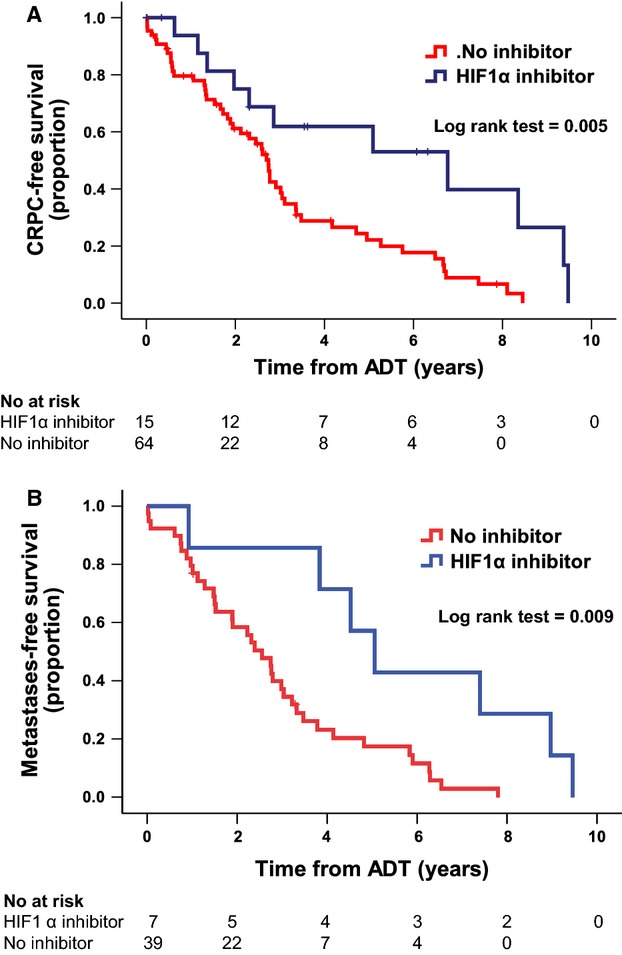
Kaplan–Meier analyses of time to CRPC and time to metastases. The survival curves for CRPC-free survival (A) and metastases-free survival (B) versus the time from starting androgen deprivation therapy are significantly better for patients treated with HIF1*α* inhibitors. Fourteen men (14.3%) were excluded from the analysis as they had missing data. CRPC, castrate-resistant prostate cancer; HIF, hypoxia-inducible factor.

On a univariate analysis, men on HIF1*α* inhibitors had a 73% lower risk of developing castrate resistance compared to the men receiving no inhibitors, while a high Gleason score increased the risk by 1.4-fold (Table 2). The median time for developing CRPC (Fig. 2) in patients not on HIF1*α* inhibitors was 2.7 years compared to 6.7 years in patients using HIF1*α* inhibitors (log-rank test *P* = 0.005).

**Table 2:** Univariate and multivariate regression analyses.

		Univariate analysis			Multivariate analysis		
	No. of patients (%)	Hazard ratio	95% CI	*P*-value	Hazard ratio	95% CI	*P*-value
Time to CRPC	84 (100)						
HIF1*α* inhibitor		0.37	(0.18–0.76)	0.007	0.29	(0.10–0.78)	0.016
Gleason score		1.35	(1.04–1.74)	0.024	1.31	(0.99–1.72)	0.057
Age		0.96	(0.93–1.00)	0.014	1.00	(0.98–1.04)	0.553
PSA		1.00	(1.00–1.00)	0.370	1.00	(1.00–1.00)	0.403
Time to metastases	46 (54)						
HIF1*α* inhibitor		0.29	(0.11–0.77)	0.013	0.19	(0.05–0.76)	0.019
Gleason score		1.44	(1.03–2.01)	0.035	1.50	(1.05–2.13)	0.025
Age		0.97	(0.93–1.01)	0.220	1.00	(0.96–1.06)	0.866
PSA		1.00	(1.00–1.00)	0.947	1.00	(1.00–1.00)	0.284

CRPC, castrate-resistant prostate cancer; HIF, hypoxia-inducible factor; PSA, prostate-specific antigen. Cox proportional univariate analyses of time to castrate resistance and time to metastases are presented. Multivariate regression analyses adjusting for each of the other factors are also presented. Fourteen men (14.3%) were excluded from the analysis as they had missing data.

On a multivariate regression analysis adjusting for Gleason score, age, and PSA level at the time of starting androgen deprivation, there was a 71% reduction (HR 0.29 [CI 0.10–0.78] *P* = 0.02) in the risk of developing CRPC in patients who were on HIF1*α* inhibitors. Gleason score was an independent risk factor for development of CRPC (Table 2).

### Time to metastases

There were no differences in baseline Gleason scores between those men included in the time to metastases analysis and those excluded from it, or in those men on HIF1*α* inhibitors and those not on HIF1*α* inhibitors.

Men on HIF1*α* inhibitors and on androgen deprivation had a significantly lower risk of developing metastases (71%). A high Gleason score was a predictor of developing metastases in patients starting HIF1*α* inhibitors (Table 2). The median metastases-free survival time (Fig. 2) in men on HIF1*α* inhibitors was significantly longer than in those not on inhibitors (5.1 years vs. 2.6 years, *P* = 0.009).

There was an 81% reduction in the risk of developing metastases in men on HIF1*α* inhibitors (HR 0.19 [CI 0.05–0.76] *P* = 0.02) after adjusting for age, Gleason score, and PSA level (Table 2).

The average time from commencing HIF1*α* inhibitor use to metastases was 54 months (range 3–116 months) and the duration of inhibitor use did not have any effect on the outcomes. In order to establish if these effects were specific to the medication used, further analyses were performed on men taking either aspirin, statins, *β* blockers or an ACE-1 inhibitor. There was no significant reduction in time in metastases (HR 0.71 [95% CI 0.4–1.4] *P* = 0.3) or time to castrate resistance (HR 0.69 [95% CI 0.4–1.1] *P* = 0.2) on a univariate analysis.

## Discussion

Expression of androgen receptors has been linked with development of castrate resistance 8, and shown to be upregulated by HIF1*α* 9. Furthermore, HIF1*α* is upregulated in prostate tumors 10,11 and is a potent defensive mechanism used by tumor cells against oxidative stress or destruction by androgen deprivation, chemotherapy or radiation cytotoxicity 12. These results are supported by our previously published data, which demonstrated that overexpression of HIF1*α* increases the risk of castrate resistance and metastases in PC 4, and confirmed the importance of HIF1*α* as a molecular target in PC.

The aim of this pilot study was to investigate the effects common to the nonspecific HIF1*α* inhibitors digoxin, metformin, and ARB on the development of CRPC and metastases. Our results demonstrate a significant increase in median metastases-free survival of 2.5 years and CRPC-free survival of 4 years in men using HIF1*α* inhibitors. While the number of patients on HIF1*α* inhibitors was too small to analyze further the effects of the individual drugs, all three medications individually demonstrated similar trends toward the benefits seen above. Since all three drugs inhibit HIF1*α* expression, this observation is consistent with the suggestion that the increased survival is the result of a reduction in HIF1*α* expression. Furthermore, the side effects of these nonspecific HIF1*α* inhibitors are generally more tolerable compared with those of the commonly used chemotherapeutic agents. Thus, the use of nonspecific HIF1*α* inhibitors to delay castrate resistance and metastases is likely to be more advantageous to patients starting androgen deprivation, rather than following development of CRPC. These results, seen even in a small sample size, are in agreement with our previous data that linked HIF1*α* overexpression with decreased survival 4. Larger prospective studies are warranted to confirm our findings.

The objective of this study was to analyze the effect of HIF1*α* inhibitors as a class rather than as individual agents. All three of the agents investigated have been shown to reduce the risk of developing various cancers 13–15. Digoxin, a universal protein translation inhibitor that inhibits HIF1*α* expression in vitro and in vivo 5, reduces the risk of PC, including potentially lethal disease, by 75% 16. Metformin, on the other hand, inhibits HIF1*α* by an indirect mechanism 6, and increases apoptosis and the anti-proliferative effects of bicalutamide in PC 17. Although Bansal et al. demonstrated that there was a 14% reduction in the incidence of PC in men with type 2 diabetes 18, other studies do not provide a clear consensus on whether there is a link between these conditions. Furthermore, while some studies showed a potential link between diabetes and disease progression 19, metformin treatment of men with diabetes has been shown to improve PSA-recurrence-free survival, distant metastases-free survival, PC-specific mortality, overall survival, and development of CRPC, compared to men with diabetes not on metformin 20. Metformin also reduces the incidence of high-grade PCs and their progression 21, and confers some survival benefit by reducing metabolic syndrome in men with PC 22,23. As angiotensin 2 has been shown to increase HIF1*α* in vitro by a posttranslational mechanism 7, ARBs would be expected to reduce HIF1*α* expression. Although ARBs have been shown to reduce proliferation of PCs in vitro 24, the inhibitory effects of different ARBs on HIF1*α* expression have not been compared and should be studied further.

Although this study did not directly demonstrate that the effects of digoxin, metformin, and ARBs are mediated only via inhibition of the HIF1*α* pathway, a feature common to all three drugs is that they inhibit HIF1*α* expression in cell lines in vitro. Thus, a reasonable working hypothesis is that the main actions of these agents are via the HIF1*α* pathway. Apart from one study demonstrating HIF1*α* inhibition at 2 g daily of metformin (in keeping with the doses used by some patients in our study) 25, there are no clinical studies looking at the optimal dosage at which these medications inhibit HIF1*α*, and only preclinical studies observing the inhibition of HIF1*α* by digoxin or ARBs. This study demonstrates that there may be some benefit even at the doses usually taken by patients and these could be used as a pilot for further studies.

Many men with CRPC would also develop resistance to chemotherapy and would subsequently require additional hormonal or immunotherapeutic agents. However, the currently reported survival advantages in men started on newer hormonal or immunotherapeutic agents such as abiratarone acetate or sipuleucel-T following development of CRPC is less than a year 26. Treatment with nonspecific HIF1*α* inhibitors might also give an added survival benefit when used with the newer hormonal agents after development of CRPC. A major obstacle in designing trials using HIF1*α* inhibitors is the length of the study, since many years might elapse prior to reaching the endpoints of CRPC and metastases. Thus, the evidence from prospectively maintained databases is of great interest.

There would be several advantages in using nonspecific HIF1*α* inhibitors to reduce the risk of developing castrate resistance and metastases in patients on androgen deprivation. Because of the availability of generic brands, the costs to the consumer would be negligible compared to new cancer drugs. Furthermore, due to their widespread long-term use, their side effects are well established in a population of patients who maybe concurrently on these drugs while on ADT. However, the use of these drugs may give rise to new issues such as the use of metformin in nondiabetic patients, and potential cardiac toxicity with digoxin. The need for monitoring of drug levels and the lack of familiarity of urologists and oncologists in the routine prescription of these medications will also require further consideration prior to starting these medications for PC. In order to negate these issues, the future development of specific HIF1*α* inhibitors would be desirable.

The design of this study only identified men who underwent chemotherapy for CRPC, which may have led to an element of selection bias. Thus, the present results cannot be extrapolated to those who were not medically fit for chemotherapy, or did not survive to the point of considering it. The original indication for the use of drugs was cardiovascular disease and/or hypertension but the severity in the HIF1*α* inhibitor group, and the presence of cardiovascular disease in men not on HIF1*α* inhibitors, was not recorded. Thus, these factors could not be adjusted for in the analysis, and the results may have been influenced accordingly. In order to exclude the effects of the severity of cardiovascular disease on mortality, our aims were focussed on cancer-specific outcomes, which did not include mortality. Another limitation of the study is that the medication history was recorded at the start of ADT, and it is possible that the drug doses or compliance with these medications may have changed during the course of therapy. In addition, these findings could be influenced by sample bias due to the small numbers available for this study. Therefore, larger prospective studies should be designed to investigate these findings further.

## Conclusions

Nonspecific HIF1*α* inhibitors appear to increase the progression-free survival and reduce the risk of developing CRPC and metastases in patients on continuous ADT. Larger prospective studies are warranted to confirm these findings. Future trials may be able to utilize these nonspecific inhibitors, pending the development of agents specifically targeting HIF1*α*.
